# Supplementary material for: Molecular Analysis of Fetal and Adult Primary Human Liver Sinusoidal Endothelial Cells: A Comparison to Other Endothelial Cells
Source: Int J Mol Sci. 2020 Oct 21;21(20):7776. doi: 10.3390/ijms21207776 (PMC7589710; doi:10.3390/ijms21207776)
Supplement: Supplementary file 1 [file ijms-21-07776-s001.zip › Supplementary material.docx]

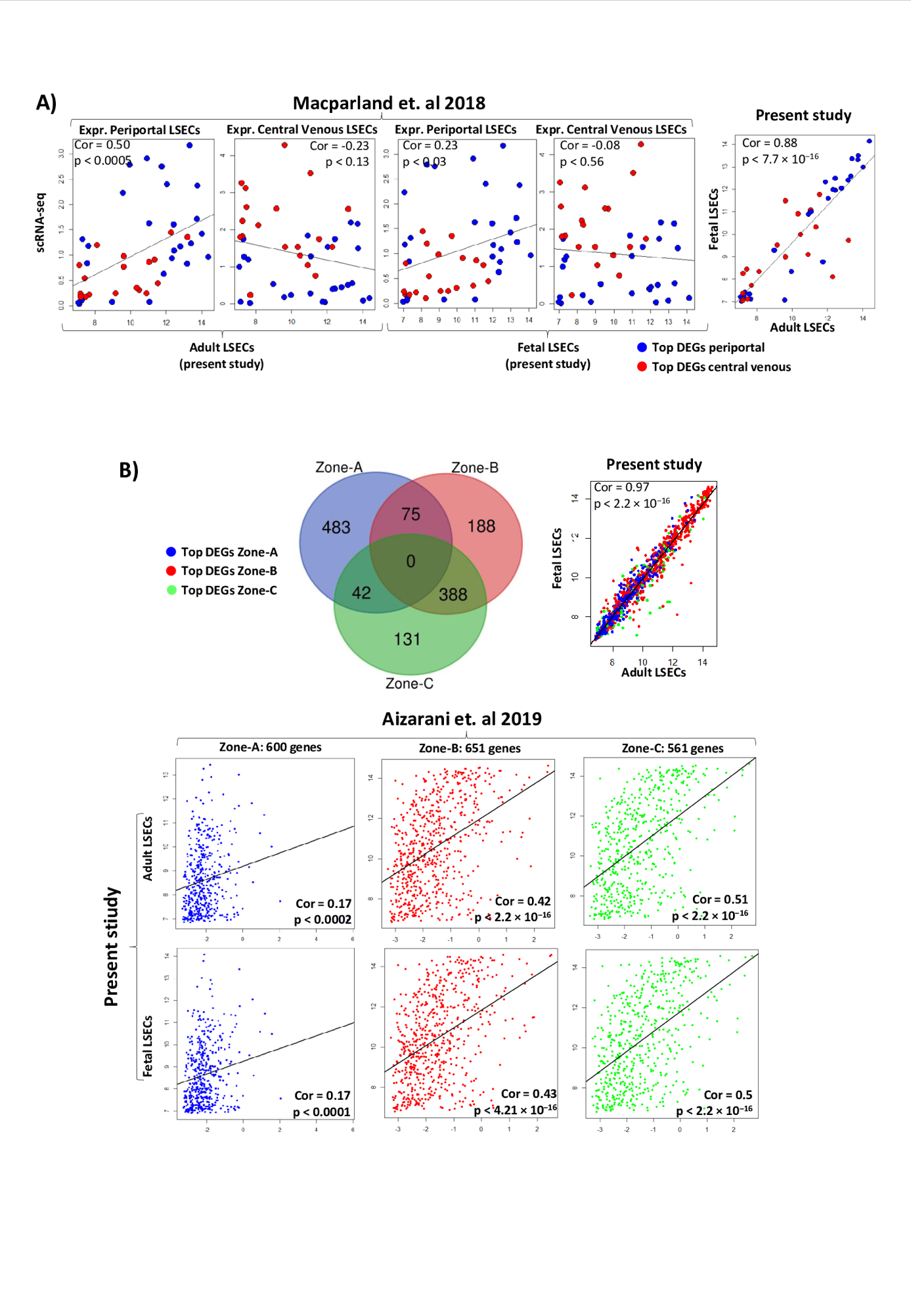


**Supplementary Figure 1.** Comparison of primary liver sinusoidal endothelial cells (LSECs) with single cell data: **A)** Correlation of MacParland et. al 2018 reported genes between (left) present study LSECs (adult and fetal) with MacParland et. al 2018 identified LSECs, and (right) a-LSECs and f-LSECs. **B)** (Left) Venn diagram of differentially expressed genes identified for different zones of LSECs reported in Aizarani et. al 2019, (right) correlation between a-LSECs and f-LSECs of all LSECs genes reported by Aizarani et. al 2019. **C)** Correlation of DEGs in different zones of Aizarani et. al 2019 with primary a-LSECs and f-LSECs.


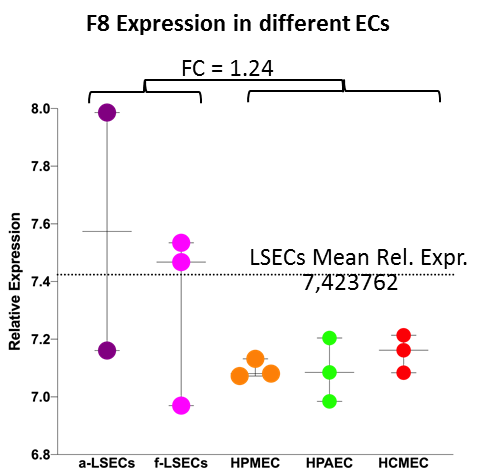


**Supplementary Figure 2.** Relative F8 expression in LSECs (adult and fetal), HPMEC, HPAEC and HCMEC.


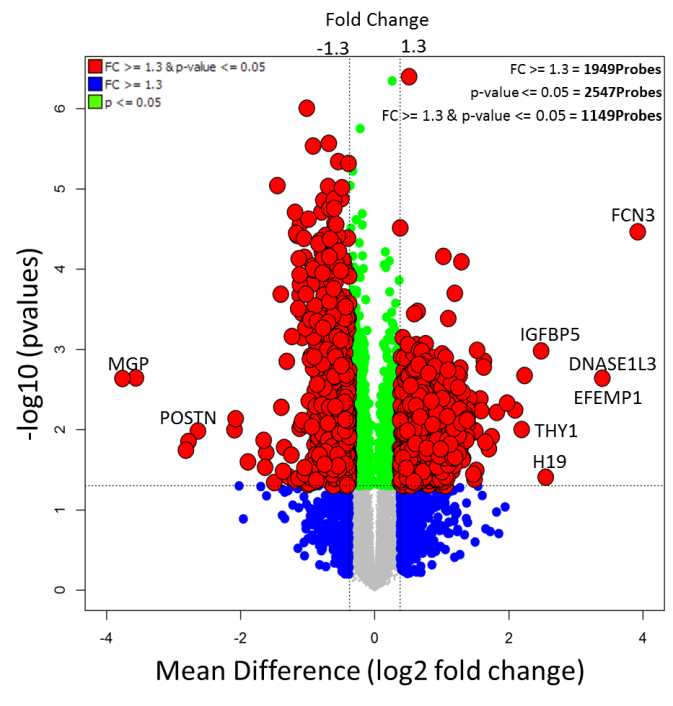


**Supplementary Figure 3.** Volcano plot between f-LSECs and other fetal endothelial cells (f-ECs) (HPMEC, HCMEC & HPAEC). Red = Genes with foldchange above 1.3 and p-value below 0.05, Blue = Genes with foldchange above 1.3, and Green = Genes with p-value below 0.05


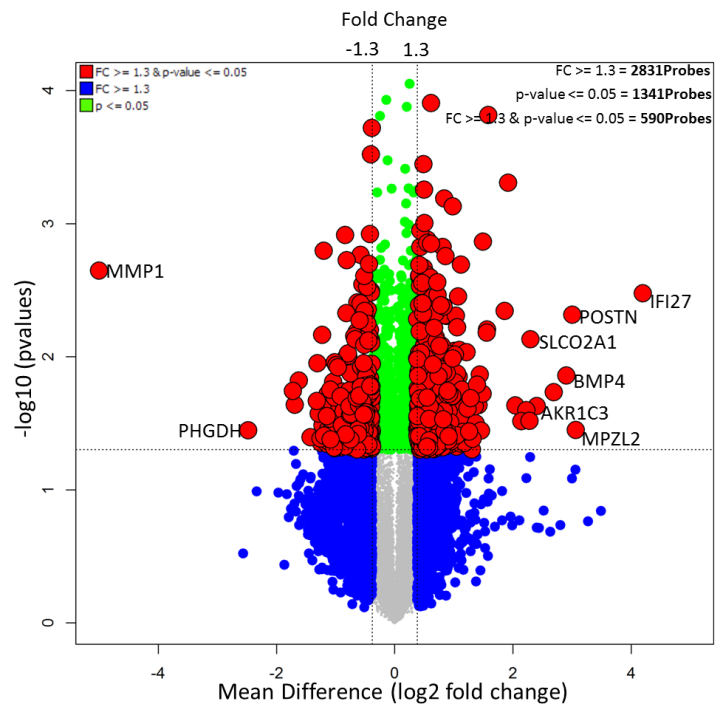


**Supplementary Figure 4.** Volcano plot between a-LSECs and other f-LSECs. Red = Genes with foldchange above 1.3 and p-value below 0.05, Blue = Genes with foldchange above 1.3, and Green = Genes with p-value below 0.05
